# Population status, foraging ecology and activity pattern of golden jackal (*Canis aureus*) in Guangua Ellala Forest, Awi Zone, north west Ethiopia

**DOI:** 10.1371/journal.pone.0233556

**Published:** 2020-05-29

**Authors:** Tilahun Gashe, Mesele Yihune

**Affiliations:** 1 Department of Biology, Debre Markos University, Debre Markos, Ethiopia; 2 Department of Zoological Sciences, Addis Ababa University, Addis Ababa, Ethiopia; Sichuan University, CHINA

## Abstract

A study on population status, foraging ecology and activity pattern of golden jackal (*Canis aureus*) was conducted from October 2017 to August 2018. Data was collected through direct observation (total count, focal and scan sampling) and faecal dropping analysis. Data was analyzed using descriptive statistics and compared with Chi-square test, t test and one way ANOVA. The result indicated that the average number of golden jackal in the study area was 65 during the wet season and 83 during the dry season. There was a significant difference in the population size of golden jackal between the wet and the dry season (t = 38.13, df = 1, P<0.05). The mean pack size ± SD were 4±1.19 and 4.5±1.3 during the wet and the dry seasons, respectively. Golden jackal was observed feeding mostly on rodents and plant materials. The food items consumed were significantly differed (χ^2^ = 20.33, df = 5, P< 0.05) between both seasons. They were mostly active during early morning (6:00–8:00) and late afternoon (16:00–18:00). The overall status of the current population does not appear in an immediate danger. However, there are many conservation problems that could affect the species in the future in the area. Therefore, appropriate conservation measures should be taken in to consideration to protect golden jackal and create suitable habitat.

## Introduction

Golden jackal is a medium sized canid in the genus *Canis*. It is distinguished by its basic golden coat color that varies from pale creamy yellow to dark tawny [1]. On average, the body weight of adult male and female golden jackal is estimated as 6.6 kg and 5.8 kg, respectively [2]. Golden jackals are habitat generalists. They are abundantly distributed in cultivated land, shrubland, desert, semi arid area, marsh land, forest and near human settlements [3,4]. They are distributed in many areas of Africa, Asia and Europe [5]. This confirms that the species adapts to heterogeneous environmental conditions and their efficiency in adapting to diverse habitats [6]. It is widely distributed in the Ethiopian highlands as well [7]. In addition, it is fairly common species, that is found at high densities in suitable areas and able to thrive even close to human settlements[8]. Over its entire range with the exception of protected areas like national parks and sanctuaries, the golden jackal population is continuously declining in its range. The main threat to the species comes from the reduction in forest cover and food scarcity [9]. It is a versatile predator and opportunistic feeder [10]. It mostly feeds on small mammals, mainly rodents [11], ungulates [12], cattle and poultry [13], occasionally birds and invertebrates. It is also scavenges, feeding on carcasses [14]. Generally, depending on the availability, they use variety of food items [15].

Guangua Ellala Forest is one of the forests in Awi zone. People around the forest use it for their subsistence livelihood including fuelwood and grazing. Many wildlife species in the area are continuously declining due to lack of effective conservation. The golden jackal is one of the species found in this forest and affected by the anthropogenic factors. However, no study has been conducted on the species in Guangua Ellala Forest. For effective conservation of a certain wildlife species, the presences of basic information is essential. Therefore, the present study was conducted to generate information on the current population status, foraging ecology and activity pattern of the golden jackal in Guangua Ellala Forest.

## Materials and methods

### Study site

**Fig 1:**
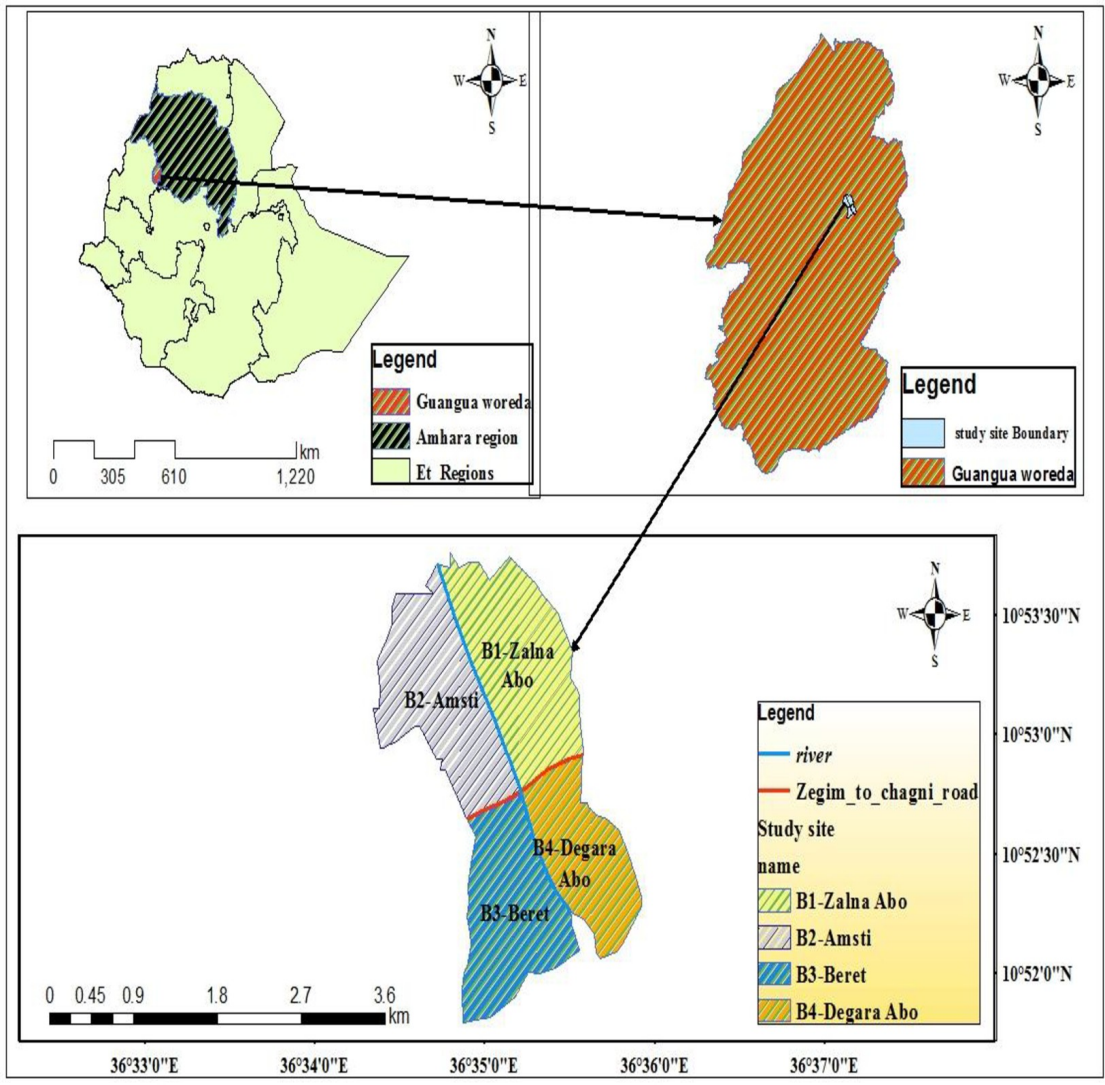
Map of Guangua Ellala Forest, Amhara region, Ethiopia showing study area of golden jackal (Arch GIS 10.4).

Guangua Ellala Forest is located in Amhara Regional State, Awi Administrative Zone. It lies at latitude 36°35΄ 0΄΄ N to 36° 36΄ 0΄΄ N and longitude 10°52΄ 0΄΄ E to 10° 53΄ 30΄΄ E (Fig 1). It is found 525 km away from Addis Ababa, the capital of Ethiopia. The area is under protection since 1981 by Guangua woreda. It covers a total area of 63.71km^2^.The altitude ranges from 1580–2150 m asl [16].

The area is rich in water resources. Some of these are Brandi, Benbela river, Kusiney river, and Kulkul river. The soil type is reddish brown loam soil [16]. The climate of Guangua Ellala Forest varies from season to season. It is cold and dry during winter (September to April), and hot and wet during summer (May to August). The climate of this forest classified as Woina Dega Zone (1500–2400 m asl). The mean annual minimum temperature was 16°C and mean annual maximum temperature was 22°C. The mean annual rainfall in the area is 1,650 mm which ranges from 1500-1800mm and the rainfall is most prominent during May, June, July and August [16].

There are numerous plant species residing in the study area. Some of the most common plant species are *Cordia africana*, *Acacia tortillis*, *Haginia abyssinica*, *Acanthus senni*, *Croton macrostachyus*, *Ficus vasta*, *Ficus sur* and *Prunus africana*. The vegetation is basically afromontane type. The area also harbours different species of mammals such as golden jackal, wartog (*Phacochoerus africanus*), civet (*Civettictis civetta)*, spotted hyena (*Crocuta crocuta)*, serval (*Leptailurus serval*), bush pig (*Potamochoerus sarvatu*), common duicker (*Sylvicapra grimmia*), rock hyrax (*Procavia capensis*), crested porcupine (*Hystrix cristata)*, and Abyssinian hare (*Lepus habessinicus)* as well as several species of small rodents [16].

### Methods

#### Population census

To estimate the population size of golden jackal, total count method was employed during dry and wet seasons following [17] and [18]. The study area was classified in to four different blocks (Block 1 (17 km^2^), Block 2 (16.71 km^2^), Block 3 (15.8 km^2^) and Block 4 (14.2 km^2^)) based on natural and artificial boundaries. During counting, information like group size, habitat type, age and sex were recorded [19]. Animals were treated as a member of the same group if the separation distance is approximately less than 50m [20]. Census was conducted during early morning (6:00–8:00) and late afternoon (16:00–18:00) when animals are active using unaided eyes and/or 12x40 binoculars. Jackals were classified as juvenile, subadult and adult according to their body size and coat colour [19]. Two trained people were assigned in each block for counting. Counting was carried out at the same time to avoid double counting. Each block was efficiently surveyed with the traind people along transect lines.

#### Foraging ecology

Foraging ecology of golden jackal was studied using faecal dropping analysis. A total of 96 feacal samples were collected from different sample areas. Faecal samples were identified from other sympatric carnivores by using shape, colour and ingested hair [21]. During collection, information including date of collection, time of collection, habitat and location were recorded. The collected faecal samples were sun dried, grounded in mortar and washed in 1mm sieve using hot water to separate prey components and other indigestible remains, such as hair, bone, teeth, bird, sheep wool, grass and fruits. The separated hairs were washed with acetone, dehydrated in 98% ethanol, dried on filter paper and observed macroscopically by considering form, length and color [21]. Direct observation was also made to determine foraging strategy of the study animal.

#### Activity patterns

To study the activity patterns of golden jackal, a scan sampling method was employed [22]. During scan sampling, individuals were recorded as performing one of the following behaviors on the standardized data sheet; foraging, moving, resting, playing, aggression, grooming, sexual activity and other [23,24]. Focal individual was randomly selected by stratifying based on age and sex. When the focal animal was in a group, the dominant activity in the group was recorded at the beginning of the observation. Observations were carried out for 5 min at an interval of 15 min from 06:00–18:00 h; activities displayed and durations were continuously recorded using a stop watch [23].

### Data analysis

Data were analyzed using SPSS software. Descriptive statistics was used to analyze population size of the golden jackal and compared with t test between seasons. The age and sex ratio was compared using Chi-square test between seasons. Food items consumed and pack size within blocks were analyze dusing a Chi-square test between seasons. Activity patterns were also compared within a day and between seasons with a one-way ANOVA to test the differences among hourly time budget over both seasons. Tukey multiple comparison test was also used to test the actual variation between different activities (P<0.05).

## Results

### Population estimate

The result indicated that number of golden jackal recorded were 65 and 83 during wet season and dry seasons, respectively with an average number of 74 individuals. There were significant difference in the population size of golden jackals between wet and dry season (t = 38.13,df = 1,P<0.05). Among blocks, the highest number of individuals were recorded in block 4 (23.5) and the least recorded in block 1 (9.5). There was significant difference in the population size of golden jackal within each block during wet and dry seasons (t = 32.76,df = 1, P<0.05) (Table 1).

**Table 1:** Number of golden jackal recorded during wet and dry season in each block.

Season	Block				Total
	B1	B2	B3	B4	
Wet	9	20	11	25	65
Dry	10	21	30	22	83
Average	9.5	20.5	20.5	23.5	74

**Fig 2:**
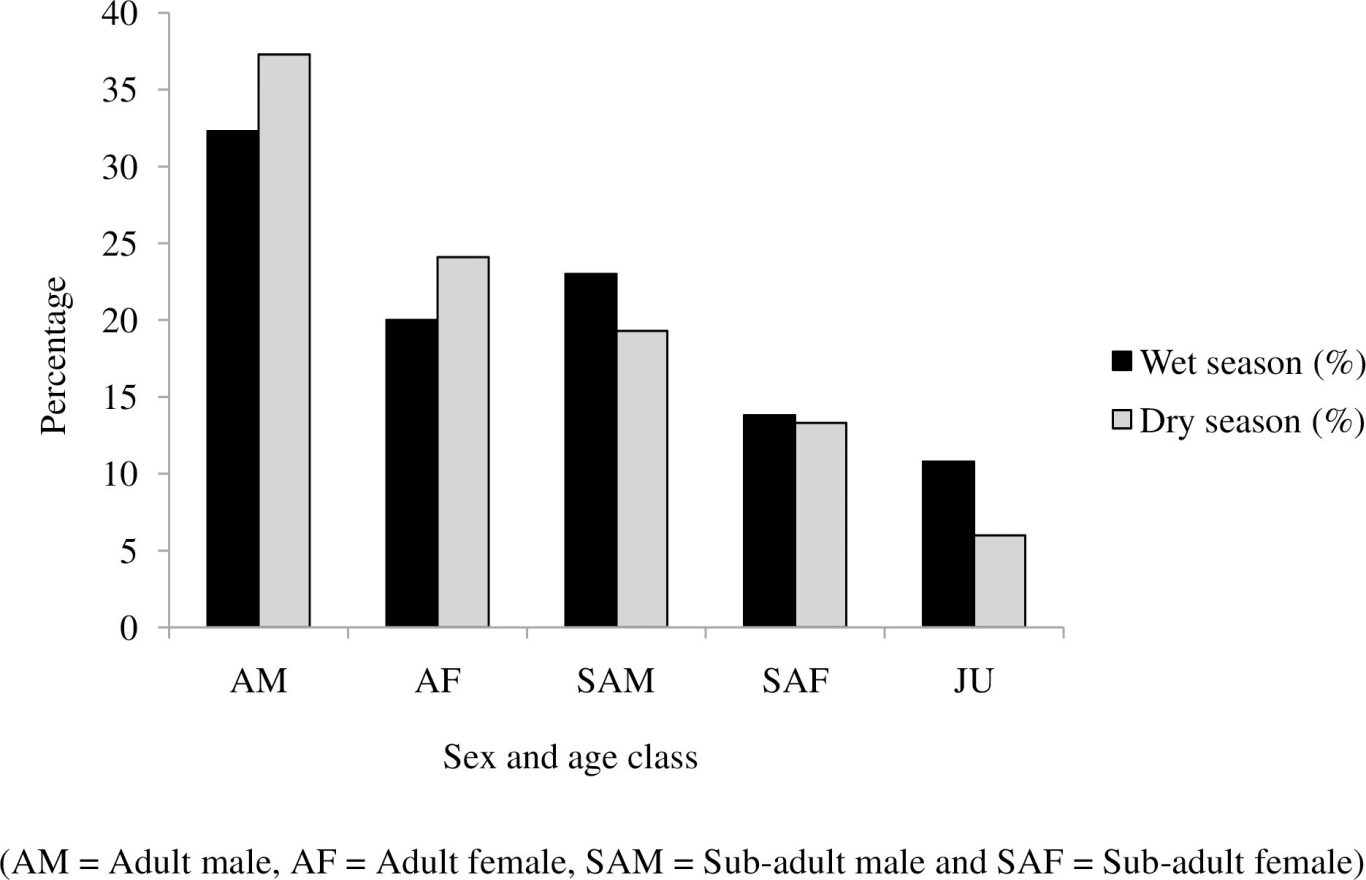
Comparison of sex and age categories of golden jackal in Guangua Ellala Forest, Ethiopia during wet and dry season.

Adult males were the highest recorded groups during the wet (32.3%) and the dry (24.1%) seasons. On the other hand, juveniles were the least recorded during wet (10.8%) and dry (6%) seasons (Fig 2).

The ratio of sub adult female to adult female was highest in the wet season(1: 0.69) while sub adult male to male ratio was lowest during the wet season (1: 0.24) (Table 2). The ratio of adult males to adult females almost similar between wet and dry season.

**Table 2:** Sex and age ratio of golden jackal during wet and dry season.

Season	Sex and age ratio			
	AM:AF	AM:SAM	AF:SAF	SAM:SAF
Wet	1:0.62	1:0.24	1:0.69	1:0.6
Dry	1:0.65	1:0.52	1:0.55	1:0.71

(AM = Adult male, AF = Adult female, SAM = Sub-adult male and SAF = Sub-adult female).

Golden jackals are mostly observed solitary or in pairs and occasionally they form a pack. Packs of up to seven indiviuals were recorded during the wet season. During the dry season, golden jackal are commonly observed in pack up to 12 individuals. Such pack didn^’^t contain more than one or two adult males but often have more than one or two adult females. The mean pack size recorded during the wet season was 4±1.19. and4.5±1.3 during the dry season. There was no statistically significant differences in the pack size between seasons (χ^2^ = 33.17, df = 1, P>0.05) (Table 3).

**Table 3:** Pack size of golden jackals during wet and dry season.

Season	Number of Individuals	Pack Size	Mean Pack Size ± SD
Wet season	9	1–7	4±1.19
Dry season	12	1–5	4.5±1.30
Mean	10.5	1–6	4.25±1.25

### Foraging ecology

**Fig 3:**
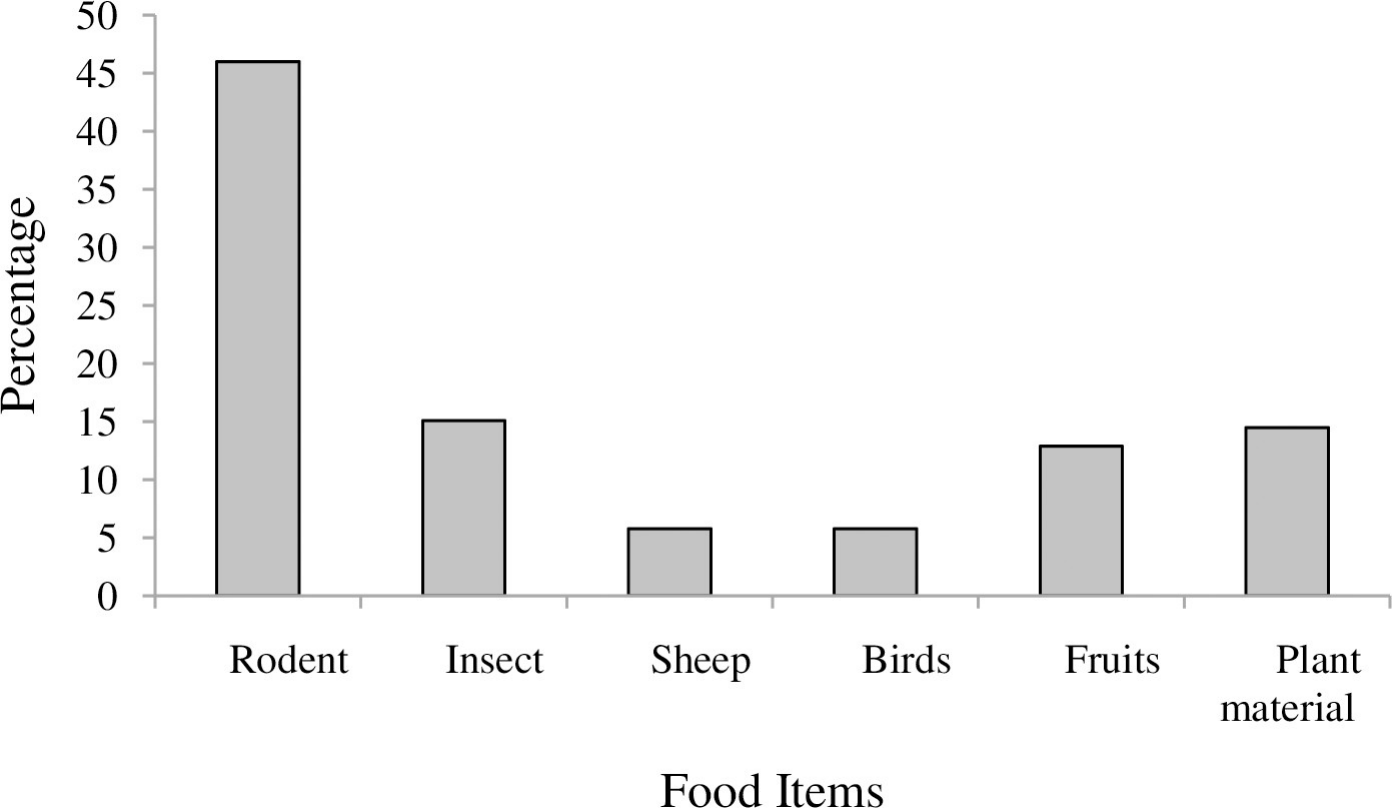
Percentage of food items consumed by golden jackal in Guangua Ellala Forest, Ethiopia.

During the study period, six different types of food items were identified from faecal analysis. Rodents (46%) were the principal food items followed by insect (15.1%) (Fig 3). Sheep (5.76%) and birds (5.76%) constituted the least food item in both season. The frequency of occurrence of food items were significantly differed (χ^2^ = 41.43, df = 5, P< 0.05).

**Fig 4:**
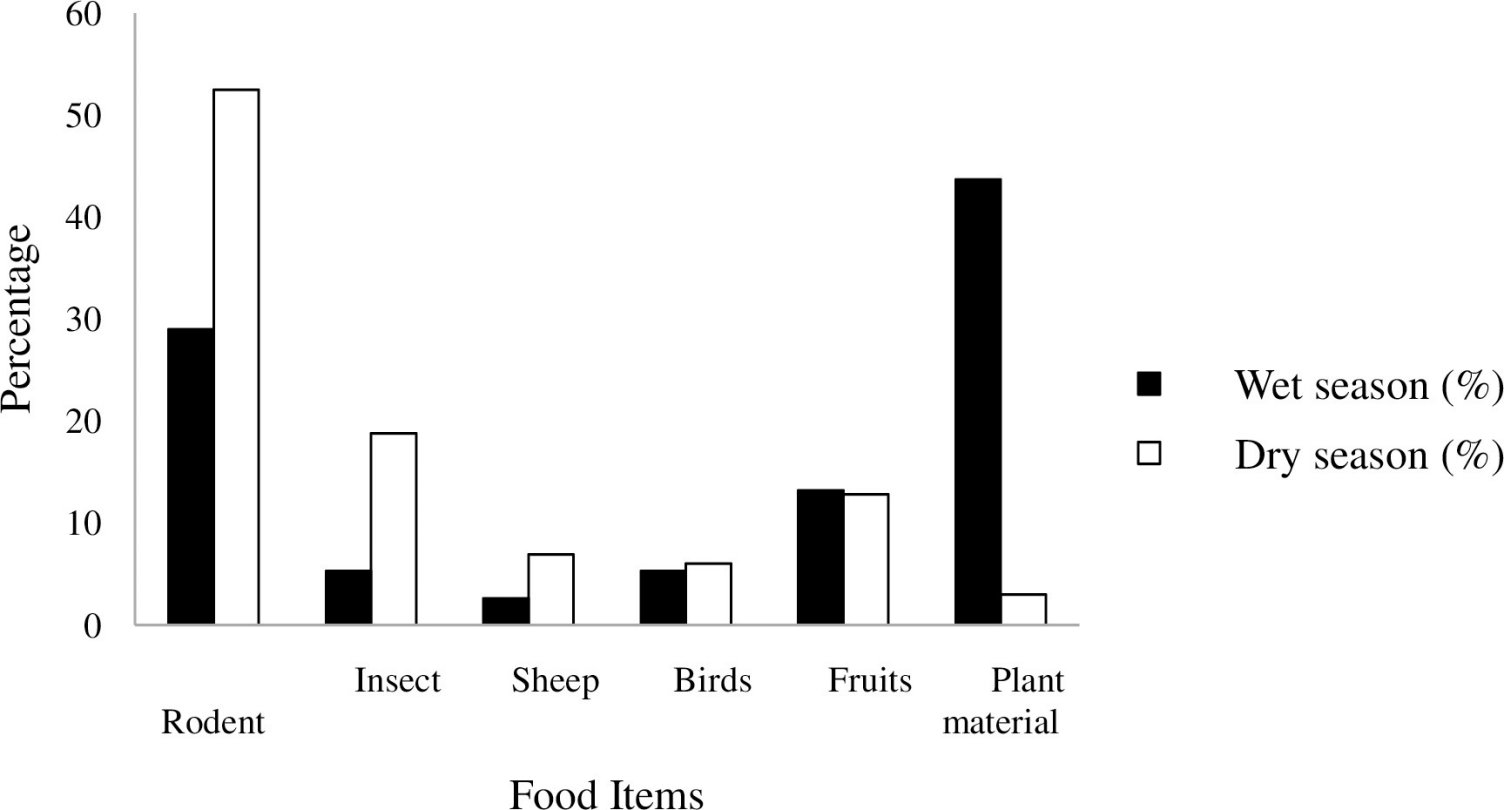
Percentage of food items consumed by golden jackal in Guangua Ellala Forest, Ethiopia during wet and dry seasons.

During the dry season, rodents (52.5%) were more important source of food. Followed by insects (18.8%). On the other hand, plant materials consisted of the highest (43.7%) proportion followed by rodents (29%) during the wet season (Fig 4). Percentage occurrence of food items was significantly differed between the wet and the dry seasons (χ^2^ = 20.33, df = 1, P < 0.05).

### Activity pattern

**Fig 5:**
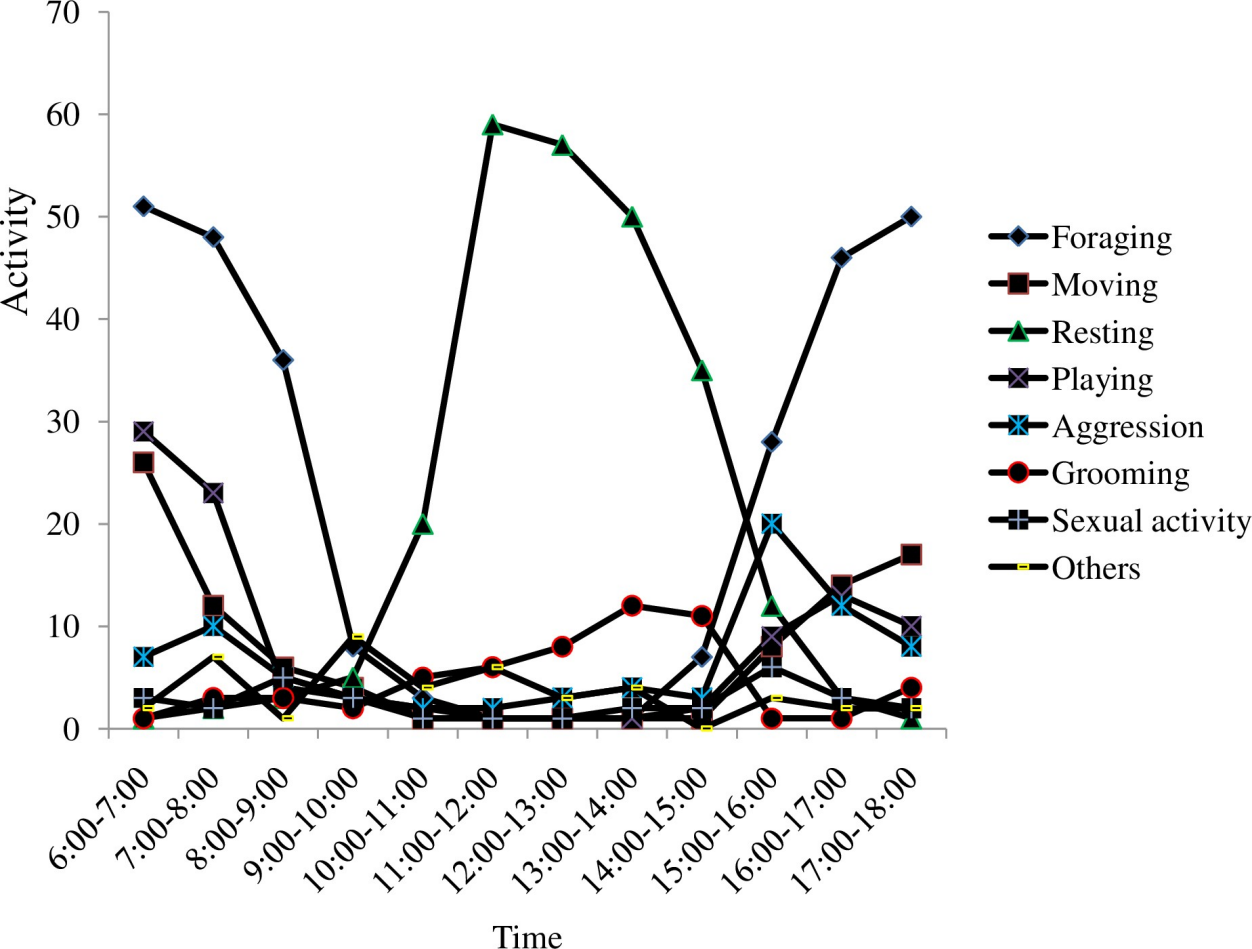
Percentage of activity patterns of golden jackal in Guangua Ellala Forest, Ethiopia.

A total of 1819 behavioral observations were recorded. Golden jackals spent more time resting (35.2%) followed by foraging (23.3%). Sexual activity (2.6%) was the least activity recorded. Sexual activity vary seasonally as it occurs seasonal bases. The activity pattern of golden jackal was significantly differed within the day (F _1817_ = 39.49, P<0.05) (Fig 5).

**Fig 6:**
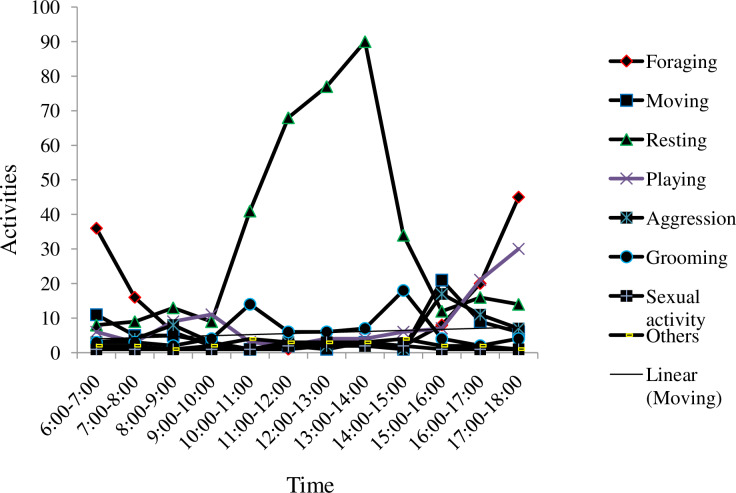
Activity time budget of golden jackal during wet season in Guangua Ellala Forest, Ethiopia.

The activity time budget indicated that there were two peaks for foraging, early morning (6:00–10:00 hrs) and late afternoon (16:00–18:00 hrs) during the wet season. On the other hand, resting was promint between 10:00 to 11:00 and 14:00 to 15:00 (Fig 6).

**Fig 7:**
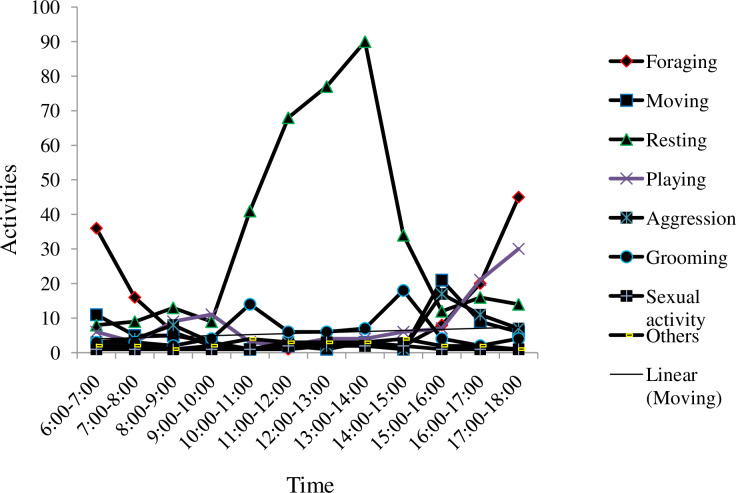
Activity time budget of golden jackal during dry season in Guangua Ellala Forest, Ethiopia.

During the dry season, the amount of time spent in foraging was decreased, with a corresponding increase in resting during the mid day between 10:00 to 14:00 hours. However, very low resting pattern was recorded between 6:00–8:00 hr and 16:00–18:00 hr (Fig 7).

## Discussion

According to the present study, the average number of golden jackals decreased from 83 (dry season) to 65 (wet season) in the study area. This could be related to opportunistic feeding behavior in search of food on fruits, vegetables and cattle predation and shelter prefernce. Usually, they prefer open habitats so, they could easily be recorded. It may also relate with difficulty for counting due to green leaves covering them during the wet season. Golden jackals occur both solitary and in groups, the advantages of living in groups is that individuals may need to be less vigilant, allowing them to use their time for foraging.

The population of golden jackal varied considerably between different blocks. This variation is most likely a result of different habitat types, food availability or accessibility and availability of den site between blocks. The number of golden jackals counted in block 4 (47) were higher than the other three blocks. Block 1 had the least number (19). The increment of the population size in Block 4 is probably related with its closeness to human settlements and it is relatively open for counting as well. The local people keep large number of sheep as their economic activity and send them to golden jackal habitat for grazing so the golden jackals spend more time in getting these sheep. This is the reason why highest number of golden jackal recorded in Block 4. In general golden jackals preferred areas where food was easily accessible and in close proximity to suitable den sites. There were many den sites in block 4 and farmlands around it. This study is agreed with the findings of Getachew[25] in Guassa Community Conservation Areas Menz, were golden jackals preferred habitats with tall and thick vegetation cover, primarily to take rest and get potential shelter and minimize threats.

The adult male and adult female sex ratio was 1:00:0.62 and it was in accordance with the expected value for most vertebrates [26]. With regard to age ratio, common perception is that, large proportion of young indicates an increasing population. However, Ramono [26] argues that this may not be necessarily true. Lower proportion of adults compared to young could indicate low survival of adults. Therefore, populations with such age structure could be declining in actual fact rather than increasing. The sex and age ratio of adult female to sub adult female was highest during wet season (1:0.69) and the ratio of adult male to sub adult male was least during wet season (1:0.24). However, the sex and age ratio of sub adult male to sub adult female were highest (1:0.71) during dry season. Accordingly, the high proportion of adult females in the present study could indicate higher degree of fertility and increase in population size of golden jackals in the study area.

In the present study, the overall mean pack size was 10.5. This could be related to the access to ample food items and suitable habitat. This study was consistant with the study of Jhalaand Moehlman [1] in Africa, where concentrated food resources resulted in aggregations of jackal. Jackal social systems appear highly flexible. This study was also agreed with findings in Israel [27], where groups of ten and twenty golden jackals aggregated around rubbish dumps. In contrast to this study, the average group size of golden jackals were 2.5 in Serengeti National Park, Tanzania [2], and average pack size in Velavadar National Park, India, was 3 [1]. Comparable data on grouping patterns are scarce but it appears that golden jackals may have been more solitary in Bale Mountains National Park than elsewhere in their range. A pair of golden jackals studied by Fuller et al, [28] in Kenya showed that the pair was in close proximity 52% of occasions. Quantitative data on jackal grouping patterns are not available from the Serengeti but it is reported to have long-term pair bonds and a high degree of behavioral synchrony between mates [29].

According to the result of the present study, golden jackals forage on 6 different type of food items. During wet season, it most frequently depends on plant material while during dry season it mostly feeds on rodents. This shows that there is a seasonal variation of food items which determine the diet of golden jackal [30]. The diet composition of jackal is dependent on a number of factors such as habitat, season, availability and encounter rate of food and the vulnerability of prey items. The present study revealed that golden jackal depends mainly on rodents, plant materials, domestic livestock, birds, fruits and seeds of a few plants for its diet. The study confirmed the opportunistic behavior of the golden jackal and its ability to adapt to various conditions in the field [30]. Similarly, the major sources of jackal diet were small mammals and plant materials at the Peljesac Peninsula in Croatia [31]. A higher frequency of small mammals in the jackal diet has been reported from India and Hungary [32].This may be related with the accessibility and ease of capture.

The occurrence of rodents in the faeces of golden jackal accounts 29% during wet season and 52.5% during dry season. This was an indication that rodents were the preferred and available food items in Guangua Ellala forest during dry season than wet season. Similarly, rodents were the principal food source as measured by incidence of occurrence. Their bones and teeth were found in 62% from Ishurdi and 56% from Mirzapur [4]. Rodents are a significant prey base for golden jackal in the study area. Similarly, Lanszki et al. [12] also found that the major food items in southern Greece were small mammals. High consumption of small mammals was also found in numerous studies in Asia [4], in Africa [33] and in European agricultural areas[14]. The frequency of rodent prey was high in faeces of golden jackals. Similar study in the agro-ecosystem of Bangladesh revealed that rodents were the most common prey in the faeces of golden jackals [4].

Plant materials were a second most important food item jackal the diet of jackal. This finding is inline with a study in Hungary, where the frequency occurrence of plant materials was found next to small mammals [12]. Plant materials such as leaves of grass species, barley and vegetables mainly carrot were also identified in faeces of golden jackal. The frequency occurrence of plant materials was high in faeces collected around settlements. This indicated that golden jackal used easily accessible materials around their den site to subsidize when there is shortage of rodent prey. Nadem et al [30] reported seeds contributed 14.7% of diet, which was relatively similar with this study. It is also similar with the study of Jaeger et al. [4] reported as plant material was found in 17% of the faeces.

Sheep occurred at a frequency of 5.6% in the faeces of golden jackal. It was more frequent during the dry season (6.9%) compared to the wet season (2.6%). The overall occurance compared to other items was low. A study conducted in Guassa Community Conservation area, Ethiopia also indicated that sheep had lower contribution as a diet of jackal [25]. The frequency of occurrence of sheep was highest during dry season. This could be related with local peoples might not guard their domestic livestock especially sheep during this season. Therefore, there was highest probability and opportunity to be preyed by golden jackal.

The activity pattern of golden jackal indicated that they were mostly active during early morning 6:00–8:00 and late afternoon 16:00–18:00. They spent on resting from 10:00–15:00 resting. As Gupta et al, [34] reported, golden jackals were spending most of the time on resting. They also show foraging, playing and grooming activities during day time. Golden jackals spent most of their time on resting followed by foraging relative to other activities conducted during the dry season. This could be related with high diurnal temperatures during the dry season and need of shelter to avoid water loss [35].

## Conclusion

The overall population status of golden jackal doesn^’^t appear in immediate danger in the study area. However, there are anthropogenic problems that could be considerd as threat to the animal. These include livestock grazing, firewood collection and agricultural encroachment. As result, habitat quality is deteriorating from time to time. Therefore, special attention should be given to conserve not only the golden jackals but also other wildlife in the forest.
